# Placenta Accreta in a Woman with Childhood Uterine Irradiation: A Case Report and Literature Review

**DOI:** 10.1155/2019/2452975

**Published:** 2019-11-05

**Authors:** Nanayo Sasagasako, Hirohiko Tani, Yoshitsugu Chigusa, Shingo Io, Haruta Mogami, Junzo Hamanishi, Akihito Horie, Eiji Kondoh, Yukiyasu Sato, Masaki Mandai

**Affiliations:** Department of Obstetrics and Gynecology, Kyoto University Hospital, Kyoto, Japan

## Abstract

The pregnancies of childhood cancer survivors who have received uterine irradiation are associated with a high risk of several obstetrical complications, including placenta accreta. The present case was a 26-year-old pregnant woman with a history of myelodysplastic syndrome treated with umbilical cord blood transplantation following chemotherapy and total body irradiation at the age of 10. Despite every possible measure to prevent preterm labor, uterine contractions became uncontrollable and a female infant weighing 892 g was vaginally delivered at 27^+4^ weeks of gestation. Under the postpartum ultrasonographic diagnosis of placenta accreta, we selected to leave the placenta *in situ*. Although emergency bilateral uterine artery embolization was required, complete resorption of the residual placenta was accomplished on the 115^th^ day postpartum. Our experience highlighted the following points. (1) The expectant management of placenta accreta arising in an irradiated uterus may not only fulfill fertility preservation, but may also reduce possible risks associated with cesarean hysterectomy. (2) Due to extreme thinning of and a poor blood supply to the myometrium, reaching an antepartum diagnosis of placenta accreta in an irradiated uterus is difficult. (3) The recurrence of placenta accreta in subsequent pregnancies needs to be considered after successful preservation of the uterus.

## 1. Introduction

Due to advances in cancer treatment modalities, including radiotherapy, the number of childhood cancer survivors (CCS) is increasing. As a result, significant numbers of CCS have reached their reproductive ages and wish to have their own children. Nevertheless, most of the CCS who have received irradiation to fields involving the ovary and uterus in their childhood have reduced fertility mainly due to premature ovarian failure [1]. Moreover, even if they become pregnant, their pregnancy courses may be hampered by several complications, such as preterm labor, fetal malposition, and low-birth weight infants [2, 3], which are attributable to myometrial fibrosis causing a reduced uterine volume and distensibility as well as vascular damage resulting in a reduced uterine blood supply [4].

Placenta accreta, which is defined as the myometrial invasion of placental villi without intervening decidual tissue, is one of the major causes of serious postpartum hemorrhage leading to significant maternal mortality. According to the depth of placental invasion, placenta accreta is divided into three types: placenta accreta, in which placental villi attach directly to the myometrium; placenta increta, in which placental villi extend into the myometrium; and placenta percreta, in which the villi penetrate through the myometrium to the uterine serosa and may invade adjacent organs [5]. The underlying etiology of placenta accreta is considered to be a failure in normal decidual formation due to a deficient endometrium. Since uterine irradiation can induce endometrial injury [4], pregnancies after uterine irradiation may be complicated by placenta accreta. Indeed, a history of uterine irradiation is listed as one of the risk factors predisposing women to placenta accreta [5]. However, since there have only been a few cases of placenta accreta reported in women with a history of uterine irradiation, an optimal management strategy has not yet been established.

We herein present a case of placenta accreta that developed in a woman with prior total body irradiation (TBI) during her childhood. The placenta was left *in situ* after the delivery and expectant management was instituted. Although emergency bilateral uterine artery embolization (UAE) was required to control massive uterine bleeding on the 10^th^ day postpartum, the residual placenta was completely resolved by the 115^th^ day postpartum. We also reviewed the literature describing similar cases to approach an optimal management strategy against this condition.

## 2. Case

The present case was a 26-year-old para 0, gravida 0, woman with a history of myelodysplastic syndrome (MDS) at the age of 10. She was treated with umbilical cord blood transplantation (CBT) following chemotherapy with cytarabine (3.4 g for 2 days), cyclophosphamide (60 mg/kg/day for 2 days), etoposide (150 mg/m^2^/day for 7 days), and mitoxantrone (5 mg/m^2^/day for 5 days) as well as 12 Gy of TBI. Complete remission with 100% donor chimerism was confirmed on the 90^th^ day after CBT. Her pubertal development progressed normally, culminating in menarche at the age of 13 and a final height of 162 cm. Although she only had menstrual bleeding a few times every year, exogenous estrogen was not administered because her serum estradiol level remained detectable in periodic blood sampling (Table 1).

At the age of 20, the patient married and visited our fertility clinic. She was oligomenorrheic with hypergonadotropic hypogonadism (serum LH 12.4 mIU/ml, FSH 28.9 mIU/ml, and E_2_ 12.2 pg/ml). The extremely low level of serum anti-Mullerian hormone (AMH, 0.01 ng/ml) suggested a reduced ovarian reserve due to her history of chemotherapy and TBI. Furthermore, a small uterus measuring only 48.3 mm in length was noted on the ultrasonographic scan. After nearly four years of unsuccessful timed intercourse, which was considered to be mainly attributable to oligo-ovulation, she decided to undergo *in vitro* fertilization (IVF). Since three courses of embryo transfer failed to result in pregnancy, she discontinued fertility treatment at the age of 25.

**Figure 1:**
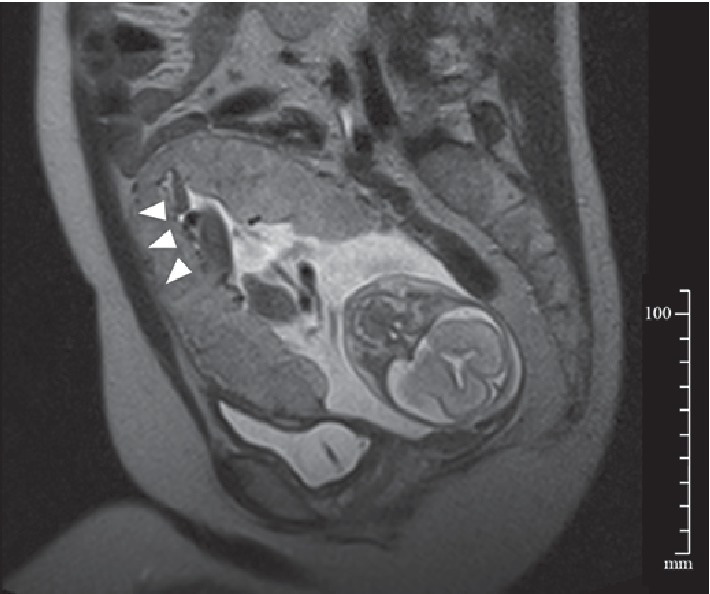
Sagittal T2-weighted magnetic resonance image of the uterus at 22^+4^ weeks of gestation. The myometrium is extremely thin throughout the uterus with partial bulging of the anterior uterine wall (arrowheads).

Two months after the cessation of fertility treatment, when the patient was 26 years old, she naturally conceived and returned to our obstetric clinic at 6^+2^ weeks of gestation. At 13^+6^ weeks of gestation, prophylactic Shirodkar cerclage was performed as the uterine cervix had shortened to 20 mm and oral nifedipine (40 mg/day) was started for tocolysis. Despite these treatments, her cervical length continued to shorten due to frequent uterine contractions. Therefore, continuous intravenous ritodrine hydrochloride was added from 16^+3^ weeks of gestation. At 17^+3^ weeks of gestation, emergency McDonald cerclage was placed at the periphery of the previous Shirodkar suture due to painless prolapse of the amniotic sac. The dose of oral nifedipine was increased to 60 mg/day and continuous intravenous magnesium sulfate was added. At 22^+4^ weeks of gestation, the patient underwent magnetic resonance imaging (MRI) scan to assess precise state of the pregnant uterus, revealing an extremely thin myometrium with only 2 mm in thickness throughout the uterus. Partial bulging of the anterior uterine wall was also noted, implying the presence of placenta accreta (Figure 1). In ultrasonography, however, we could not find any characteristic findings of placenta arcreta such as placental lacunae, loss of hypoechoic space, and abundant vascularity in the myometrium. Fetal well-being and uterine contractions were screened by daily cardiotocography. Fetal growth and amniotic fluid volume were also monitored by ultrasonography twice a week.

At 27^+1^ weeks of gestation, the passage of amniotic fluid through the uterine cervix was noted. Two doses of betamethasone (12 mg) were administered intramuscularly 24 h apart. Intravenous flomoxef (1.0 g) was given every 12 h to prevent intrauterine infection, and the dose of oral nifedipine was increased further to 160 mg/day. At 27^+4^ weeks of gestation, uterine contractions became uncontrollable. After the cerclage was removed, a female infant weighing 892 g (small for gestational age) was vaginally delivered with Apgar scores of 5 and 6 at 1 and 5 min, respectively. Under the suspicion of placenta accreta raised by the MRI finding, spontaneous placenta expulsion was awaited instead of exerting traction on the umbilical cord. The placenta remained *in situ* for more than one hour. An ultrasonographic scan revealed that the placenta was still tightly attached to the uterine wall with significant blood flow from the myometrium into the attached placenta, leading to the diagnosis of placenta accreta. Since uterine bleeding was minimal at that time and the patient expressed her desire to preserve fertility, we selected to leave the placenta *in situ*.

**Figure 2:**
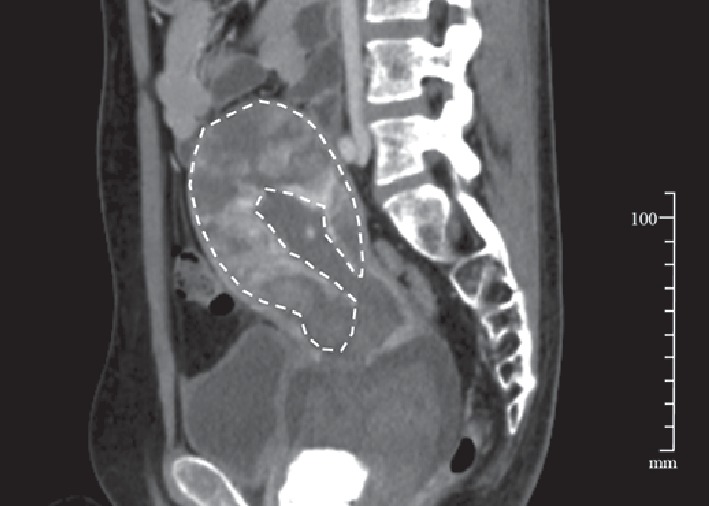
Sagittal contrast-enhanced computed tomographic image of the uterus on the 10^th^ postpartum day. The retained placenta is circumscribed by a dashed line. Note that the placenta is enhanced by contrast medium in a patchy manner.

**Figure 3:**
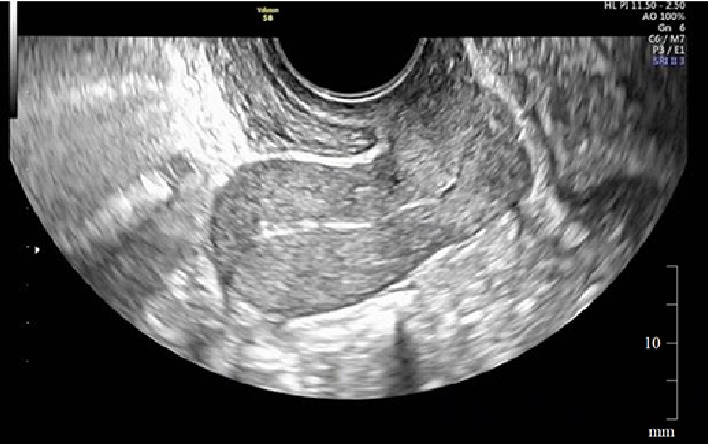
Sagittal ultrasound image of the uterus on the 115^th^ postpartum day. The placenta has become undetectable in the uterine cavity. Note that myometrial thickness has returned to normal.

On the 10^th^ postpartum day, intractable uterine bleeding of more than 300 ml per hour suddenly occurred. Contrast-enhanced computed tomography (CT) revealed the persistence of a significant placental blood supply (Figure 2). Six units of red blood cells were transfused and emergency bilateral uterine artery embolization (UAE) was conducted. Uterine bleeding subsequently subsided and she was discharged from the hospital with the whole placenta left *in situ* on the 28^th^ postpartum day. Her condition was followed up weekly at the outpatient clinic. Her serum hCG levels became lower than the cut-off value on the 87^th^ postpartum day, and complete resorption of the residual placenta was ultrasonographically verified on the 115^th^ day postpartum (Figure 3). 

The neonate suffered from respiratory distress syndrome and was given surfactant immediately after the birth. She also had duodenal stenosis with annular pancreas, which required an operation at the age of 8 days. Her postoperative course was uneventful. Respiratory care became unnecessary at the age of 50 days and she was discharged home at the age of 116 days, when her body weight reached 2922 g.

## 3. Discussion

According to our Pubmed search using “placenta accreta” and “total body irradiation” or “radiotherapy” as key words, only five cases of placenta accreta, including the present case, have been reported in women with a history of radiotherapy involving the uterus [6–9] (Table 2). The degree of uterine damage depends not only on the total irradiation dose, but also on a patient's age at the time of radiotherapy [4]. The irradiation doses in these five cases (8.75–72 Gy) exceeded the threshold value of 4 Gy, below which patients were reported to be free of the risk of subfertility [10]. Notably, radiotherapy was given before menarche in all the five cases except for case 3, which is consistent with the fact that pre-pubertal uterus is more radiosensitive than post-pubertal uterus [4]. In contrast, the risk of early ovarian functional loss becomes greater with increasing age at the time of radiotherapy [11]. In case 3, post-pubertal irradiation deprived the patient of her ovarian function, necessitating oocyte donation to become pregnant, whereas in the other four cases, the patients naturally conceived, indicating that their ovarian function had been preserved post-irradiation. In summary, women whose uteri and ovaries were irradiated during the pre-pubertal period may preserve their potential to conceive naturally; however, their pregnancies are associated with a high risk of developing placenta accreta.

Cesarean hysterectomy is currently the gold standard treatment for placenta accreta, but is associated with high rates (~40%) of maternal complications, such as massive blood loss, bladder injury, thromboembolism, and fistula formation [12, 13]. Since surgeries in previously irradiated fields have an increased risk of postoperative complications mainly due to delayed wound healing [14, 15], complication rates may be even higher if cesarean hysterectomy is performed on an irradiated uterus. Expectant management, i.e., leaving the placenta *in situ* and waiting for its spontaneous resorption, has the potential to reduce the necessity for invasive surgery, and, thus, may be preferable to cesarean hysterectomy as a strategy to manage placenta accreta that develops in women with a history of uterine irradiation. In the present case, since the patient had given birth vaginally and late postpartum hemorrhage was successfully controlled by UAE, we were able to circumvent any type of surgery, including cesarean section. Nevertheless, antenatal care and attempt at vaginal delivery in women with prior uterine irradiation need to be undertaken with great caution because these uteri are liable to rupture, as reported in cases 1 and 2, for which spontaneous uterine rupture occurred at 13 and 17 weeks of gestation, respectively (Table 2).

An antepartum diagnosis of placenta accreta allows for a planned, controlled delivery, which may minimize maternal and fetal risks [16]. Therefore, the diagnosis of placenta accreta needs to be aggressively pursued in any woman with risk factors, including uterine irradiation. In all five cases, including the present case, the diagnosis of placenta accreta was made during laparotomy or after vaginal delivery. The mainstay of an antepartum diagnosis is ultrasonography. Characteristic ultrasonographic findings associated with placenta accreta include the presence of placental lacunae, retroplacental myometrial thinning of less than 1 mm, the loss of a normal hypoechoic retroplacental zone, and hypervascularity at the bladder-myometrium interface [17]. MRI has been used for diagnoses when ultrasonography yielded equivocal results [18]. Characteristic MRI findings associated with placenta accreta include uterine bulging, heterogeneous signal intensity in the placenta, dark intra-placental bands on T2-weighted sequences, focal interruption of the myometrium, and tenting of the bladder, resulting in overall diagnostic accuracy with a sensitivity of 94.4% and specificity of 84.0% [19]. In the present case, even a retrospective review of all ultrasonographic and MRI photos did not clearly reveal any characteristic findings of placenta accreta other than uterine bulging on MRI. This paucity of diagnostic clues may be attributed to the extreme thinning of and a relatively poor blood supply to the myometrium. Therefore, in the case of pregnancy in an irradiated uterus, it is important to note that placenta accreta may be present even without characteristic imaging findings, and, thus, a delivery needs to be scheduled accordingly.

The recurrence of placenta accreta in subsequent pregnancies needs to be considered after the successful preservation of an irradiated uterus. An adequate endometrial thickness in the peri-implantation period may contribute to minimizing this recurrent risk. In women with pre-pubertal uterine irradiation, even high-dose estrogen supplementation failed to increase uterine sizes, uterine artery blood flow, or endometrial thickness [20], indicating that pre-pubertal irradiation deprives all uterine components, i.e., the myometrium, endometrium, and uterine artery, of their estrogen reactivities. This is in contrast to a uterus irradiated post-pubertally, which at least in part preserves estrogen reactivity [21]. Therefore, some measures other than estrogen supplementation were required to recover endometrial thickness in our patient, whose uterus was irradiated pre-pubertally. In 2002, promising findings were reported by Ledee-Bataille et al., who demonstrated that a treatment with the combination of pentoxifylline (800 mg/day) and vitamin E (1,000 IU/day) significantly increased the uterine size, uterine artery blood flow, and endometrial thickness of a pre-pubertally irradiated uterus [22]. This treatment needs to be investigated in the present case in order to establish whether it has the potential to prevent the recurrence of placenta accreta.

In conclusion, our experience of the present case and a literature review of similar cases have highlighted the following three points concerning placenta accreta developing in an irradiated uterus. (1) The expectant management of placenta accreta arising in an irradiated uterus may not only fulfill fertility preservation, but may also reduce the possible risks associated with cesarean hysterectomy against an irradiated uterus. (2) Difficulties are associated with reaching an antepartum diagnosis of placenta accreta in an irradiated uterus because of the extreme thinning of and a poor blood supply to the myometrium. (3) The recurrence of placenta accreta in subsequent pregnancies needs to be considered after the successful preservation of the uterus. Future studies are needed to investigate reliable methodologies for an antepartum diagnosis of placenta accreta in an irradiated uterus. Furthermore, the development of measures to prevent the recurrence of placenta accreta needs to be pursued.

## Figures and Tables

**Table 1:** Changes in serum hormone levels after menarche.

Age (years-months)	15-7	16-0	16-8	17-4	17-8	18-3	18-7	20-1	20-7
LH (mIU/ml)	104	2.7	2.2	11.8	2.2	12.8	14.6	14.3	2.8
FSH (mIU/ml)	135.7	8.2	2.6	9.4	7.9	22	9	8	3.9
E2 (pg/ml)	14.2	30.3	69	30	19.2	34.8	45.3	39.6	107.2

**Table 2:** Cases of placenta accreta after uterine irradiation.

			Age			Irradiation						
No.	Type	Primary disease	Irradiation	Pregnancy	Gravida and parity	Site	Dose	Method of conception	Cerclage	Pregnancy outcome	Treatment	Reference
1	Percreta	Clear cell sarcoma	7	23	G1P0	Pelvis	70 Gy	Spontaneous	Not described	Uterine rupture abortion (13 weeks)	Hysterectomy	[6]
2	Percreta	Chronic myeloid leukemia	5	23	G1P0	Total body	8.75 Gy	Spontaneous	Not described	Uterine rupture abortion (17 weeks)	Hysterectomy	[7]
3	Accreta	Hodgkin's lymphoma	16	31	G1P0	Right hemipelvis, Mediastinum	36 Gy, 36 Gy	Oocyte donation (twin pregnancy)	Not described	Cesarean section live birth (35 weeks)	Hysterectomy	[8]
4	Accreta	Wilms tumor	4	23	G3P1 spontaneous abortion	Whole abdomen	10.8 Gy	Spontaneous	Performed at 11 weeks' gestation	Cesarean section live birth (37 weeks)	Manual placental removal. Hemostatic sutures	[9]
5	Accreta	Myelo-dysplastic syndrome	10	26	G1P0	Total body	12 Gy	Spontaneous	Performed at 13 and 17 weeks' gestation	Vaginal delivery live birth (27 weeks)	Placenta left *in situ. *Uterine artery embolization	This case
